# Recent Advances in Design and Implementation of Satellite Gateways for Massive Uncoordinated Access Networks

**DOI:** 10.3390/s22020565

**Published:** 2022-01-12

**Authors:** Agostino Isca, Nader Alagha, Riccardo Andreotti, Marco Andrenacci

**Affiliations:** 1 M.B.I. SRL, Via F.Squartini 7, 56121 Pisa, Italy; aisca@mbigroup.it (A.I.); randreotti@mbigroup.it (R.A.); mandrenacci@mbigroup.it (M.A.); 2 European Space Agency, Research and Technology Centre (ESTEC), Keplerlaan 1, 2201 AZ Noordwijk, The Netherlands

**Keywords:** CPU/GPU architectures, E-SSA protocol, ME-SSA, MMSE, random access, spread spectrum

## Abstract

This paper provides an overview of recent results of a design, development and performance evaluation study of satellite gateways to receive and manage the traffic from a large population of uncoordinated user terminals. In particular, direct satellite access scenarios for machine-to-machine communications and the Internet of Things have been targeted. Tests were carried out in a representative laboratory environment emulating realistic system scenarios. Performance results, as presented in this paper indicate that the proposed gateway architecture, based on an efficient access protocol, is capable of managing a very high number of uncoordinated terminals transmitting short messages with a low duty cycle. The applicability of the proposed solution to both geostationary and non-geostationary satellite systems has also been examined. The key concept of the gateway is based on a novel receiver architecture that implements the linear minimum mean square error (MMSE) spread spectrum signal detection and successive interference cancellation techniques. The receiver uses features such as a multi-stage detector together with a robust preamble detection. The end-to-end solution includes also the use of a new waveform with a quasi-constant envelope at the terminal to modulate and transmit data packets to be received and detected by the gateway via a satellite link.

## 1. Introduction

In the past decade, the ever-growing demand for machine-to-machine (M2M) communications and the emerging market of the Internet of Things (IoT) has led to a high research interest and industry development of efficient protocols for collecting data from a large and uncoordinated population of user terminals via satellite.

A direct satellite access can play a key role in collecting data from remote areas for applications such as the IoT, and M2M. The majority of such applications require only a low bitrate per connection; however, there is a large population of user terminals to share the access channel, hence the use of a conventional demand assignment per connection is quite costly in terms of traffic scheduling and control messages towards the user terminals. There are also other constraints in terms of cost and energy of the user terminal that impact the viability of the proposed service. It is therefore essential to consider efficient satellite grant-free access schemes to complement the terrestrial coverage in order to succeed in the deployment of the global IoT solutions. A recent survey of technologies and standards [1] has reported opportunities that innovative solutions can offer to Satellite IoT services. An insight into recent advances in satellite based massive machine type communications has also been recently reported in [2].

The work in [3] provides an extensive review of high-performance random access (RA) techniques suitable for the return link of satellite networks. Key aspects include lower energy consumption and lower user terminal complexity, high flexibility in terms of required bandwidths and supported data rates, and asynchronous transmission of short messages with a low duty cycle. Other important design factors are minimum signaling on the forward link for terminals’ population coordination to connect remote terminals to a central entity, e.g., smart grid, sensor networks or connected cars. 

The protocol known as Enhanced-Spread Spectrum ALOHA (E-SSA) [4] turns out to meet these requirements while providing a high spectral efficiency in terms of normalized aggregated throughput as highlighted in [3]. The E-SSA protocol has been adopted as an open standard, known as the S-band Mobile Interactive Multimedia (S-MIM) [5]. Some variations of the original E-SSA protocol have also been deployed by the satellite industry in other operating frequency bands (e.g., VHF, Ku and Ka bands). An example of such evolution is known as the Fixed Satellite Interactive Multimedia (F-SIM) protocol [6] used for uncoordinated return access of broadcast interactive systems [6].

The E-SSA protocol combines the Spread Spectrum ALOHA (SSA) protocol, used by terminals at the transmitter, with an enhanced receiver based on successive interference cancellation (SIC). In the baseline E-SSA approach [4], terminals transmit Binary Phase Shift Keying (BPSK) spread spectrum bursts sharing the same time/frequency resources in a random and uncoordinated manner. The overlapping bursts are subject to multiple access interference (MAI) at the receiver. In the case of a conventional SSA receiver, the impact of partially overlapping bursts is handled to some extent thanks to the properties of the spread spectrum signals. At a low traffic load (i.e., a low probability of burst collisions), the despreading operation allows for correctly demodulating the interfering received bursts. The conventional SSA receiver is highly sensitive to power imbalances across received bursts leading to performance degradation and the loss of throughput [4]. 

The SIC mechanism, as implemented as part of the E-SSA receiver, allows the system to decode multiple overlapping bursts even at high MAC load conditions, increasing the overall aggregated throughput. On the contrary to conventional SSA receiver, the power imbalance among the received bursts improves the performance of the SIC operation leading to a higher aggregated throughput. Essentially, the E-SSA receiver is based on a sliding window memory whose size is typically around five times the length of a burst. All the bursts detected in such an interval are sorted according to the descending values of their received power and demodulated accordingly. Each correctly demodulated burst is then locally regenerated, corrected by the received timing offset, frequency and channel gain values, and subtracted from the stored samples in the memory window. 

The iterative SIC process improves the signal to noise plus interference value (SNIR) after each stage of interference removal, thus increasing the chance of correctly detecting and demodulating additional bursts within locally stored samples of the received signals. This process continues for a given number of SIC iterations, or until the point that no additional bursts are demodulated. Then, the sliding window is advanced forward and the process starts from the beginning on the new memory window. 

It is worth noting that the E-SSA scheme does not affect the transmit waveform from the user terminal, hence no additional complexity is imposed at the transmitter. The iterative cancellation process that is more computationally intensive affects mainly the receiver that is shared among a large number of users.

Despite the significant throughput enhancement offered by the E-SSA scheme (compared to the SSA), there is a theoretical performance gap between the throughput offered by the E-SSA scheme and the achievable aggregate capacity of the corresponding multiple access channel. While the E-SSA receiver uses a single user matched filter (SUMF), it is known that the use of a linear minimum mean square error (MMSE) detector can improve the aggregate throughput, or achievable capacity of spread-spectrum schemes [7,8]. This concept was further investigated in [9] and a receiver architecture based on the approximation of the linear MMSE detector at the receiver prior to successive interference cancellation was proposed. The new receiver architecture is referred to as MMSE Enhanced Spread-Spectrum Aloha (ME-SSA) [9].

Following the initial concept introduced in [9], this paper describes the outcomes of a recently completed R&D project on “Satellite Gateway Development for Massive Uncoordinated Access Networks”, referred to as the MASSIVE project [10], where the ME-SSA scheme was implemented and its performance was demonstrated in a lab environment, representing realistic satellite IoT system scenarios. While a preliminary set of results of the MASSIVE project was already reported in [2], in this paper we provide more details regarding the project objectives, key features and main outcomes, complementing previously reported results.

In particular, in the MASSIVE project the following aspects were investigated and compared to the standardized E-SSA protocol [4,5] using a lab test-bed environment:

- Increased flexibility of the air-interface, by supporting quadrature phase shift keying (QPSK) modulation and time domain pilot insertion, as opposed to the overlaid BPSK data and signaling channels as used in E-SSA,
- Reduction of the signal envelope peak-to-average ratio by implementing a quasi-constant envelope (CE) spread-spectrum waveform [11]. This feature enables a cost reduction of the transmitters by improving the power efficiency. In particular, it allows the non-linear power amplifiers to operate closer to the saturation point without distorting the transmitted signal. This scheme also provides a sharper power spectrum in the presence of non-linearity at the transmitter, hence much lower spectral side lobes, in order to better exploit the available bandwidth especially in the case of narrowband channels,
- Implementation of the MMSE receiver based on the multi-stage detector/despreader (MSD) approximation [9], which ensures a better performance when coupled with the QPSK modulation at the transmitter,
- Implementation of a more robust preamble searching and detection algorithm, in order to deal with harsher channel conditions (e.g., larger frequency offsets and Doppler rate values, typical of Low Earth Orbit (LEO) or Medium Earth Orbit (MEO) satellites scenarios).

The core activities of the MASSIVE project [10] were the implementation of the above-mentioned features. A test-bed, equipped with multiple central processing units (CPU) and graphic processing units (GPU), was developed to implement the new waveforms and the ME-SSA receiver in order to demodulate, in real-time, the transmitted waveforms from a random traffic generator representing a large population of IoT satellite terminals. 

*Notations*. Matrices are in upper case bold while scalar variables are either in upper or lower case italic. 

## 2. Scenario Overview and Organization of the Paper

**Figure 1:**
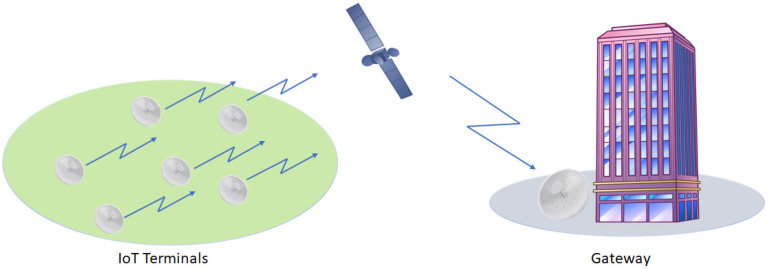
MASSIVE system scenario overview.

The target MASSIVE scenario is depicted in Figure 1, showing an uncoordinated (grant-free) population of IoT terminals which transmit a low volume traffic with a low duty cycle, a concept often referred to as sporadic transmission, towards a gateway via a satellite return link. Both geostationary and non-geo-stationary satellites were considered in this study.

The rest of the paper is organized as follows. Section 3 provides a description of the physical (PHY) layer used by each IoT terminal for the uplink transmission on the return link direction from the user terminal towards the gateway via the satellite. Section 3 also highlights the new features implemented in the MASSIVE test-bed, compared to the E-SSA based air interface [5]. 

Section 4 describes the signal receiver at the gateway that shall be able to demodulate the transmitted bursts by a large population of satellite IoT transmitters. In particular, Section 4.1 gives an overview of the receiver structure. Section 4.2 and Section 4.3 focus on the two main innovative blocks of the ME-SSA receiver which were studied, implemented and assessed during the project; the preamble searcher (PS) and MMSE detector based on MSD approximation, respectively. 

Section 5 describes the test-bed used for the validation of the results and gives the implementation guidelines to efficiently execute the most computationally demanding block, that is, the MSD receiver. 

Section 6 provides a summary of the main results and outcomes from the performed simulations, highlighting the causes of implementation loss in a real-time receiver compared to the theoretical results available from the literature and the adopted solutions to minimize such losses. 

Finally, concluding remarks and future perspectives are drawn in Section 7 and Section 8, respectively.

## 3. ME-SSA Transmitter Design and Waveform Definition

This section describes the characteristics of the ME-SSA PHY layer used for burst generation and introduces the main features of the MASSIVE waveforms. 

**Figure 2:**
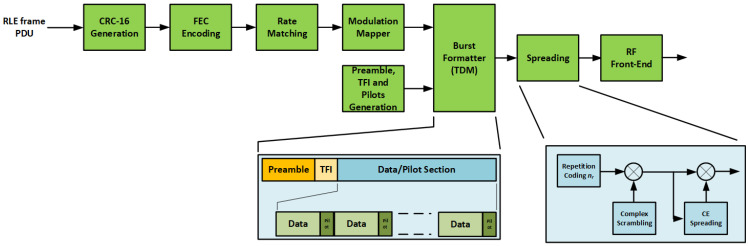
MASSIVE burst generator.

Figure 2 illustrates a high-level block diagram of the burst generator. Each burst is composed by the sequence of the following fields: preamble symbols, transmit format indicator (TFI) symbols, data symbols and periodically inserted pilot symbols. The data symbols are constructed as follows.

A cyclic redundancy check (CRC) bit sequence was appended to the incoming information bits from the upper layer. The resulting data packet was encoded by a forward error correction (FEC) turbo coding scheme and mapped into QPSK modulated symbols.

Preamble and pilot symbols were inserted as shown in Figure 2 to ease the burst acquisition and the estimation of the burst symbols timing offset, carrier frequency and phase of the received burst at the gateway.

The TFI field was used as an identifier of the transmitted waveform containing parameters such as coding rate, modulation, spreading factor and payload data size. The TFI field allows the receiver to tune the proper demodulation parameters according to the detected burst. 

In the following subsections, the impact of the two new features with respect to the conventional E-SSA burst format is discussed, i.e., the addition of the QPSK modulation (Section 3.1) and the adoption of CE spreading (Section 3.2). Finally, in Section 3.3 the MASSIVE waveforms are defined.

### 3.1. QPSK Modulation

As described in [9,12], the E-SSA waveform design was originally based on the 3GPP Wideband CDMA (W-CDMA) up-link air interface [12,13]. Common features include the FEC coding scheme (turbo code with rate 1/3), symbol spreading, as well as a physical layer control channel (PCCH), carrying known symbols for the channel estimation at the receiver. 

One of the concepts investigated in the MASSIVE project was the use of a linear MMSE detector at the receiver to increase the spectral efficiency. For a conventional receiver with a single user matched filter (SUMF), the use of BPSK modulation is known to reach the optimal capacity (constrained by the receiver SUMF structure) [7,8]; however, for a linear MMSE detector structure the BPSK modulation no longer provides the highest aggregate throughput. In the MASSIVE project, a QPSK modulation was considered for the transmit signal and a linear MMSE detector (one of its approximations) and successive decoding at the receiver to improve the aggregate throughput, as also indicated in [7,8,9].

The throughput of the linear MMSE detector decreases for a higher system loading, i.e., the ratio between the number of interfering bursts and the spreading factor [7]. For QPSK modulation, the system loading is almost halved with respect to that of BPSK modulation (assuming the same FEC code rate for both options and a low number of pilot and preamble symbols compared to the data symbols). This is due to higher signal space dimensions (almost double, ignoring the impact of the preamble and pilot symbols) for QPSK compared to that of BPSK modulated waveforms. The adoption of QPSK modulation requires a modification of the E-SSA waveform design. In particular, the quadrature multiplexing between the data and control channel was no longer supported. Instead, known pilot symbols were inserted regularly among the QPSK data symbols, as shown in Figure 2.

### 3.2. Quasi Constant Envelope Waveform

One of the objectives of the MASSIVE project was to reduce the complexity (hence the cost) of the satellite IoT terminals. The E-SSA based air interface solution such as S-MIM [5] uses conventional linear spreading where the BPSK modulated spreading spectrum signals are filtered by a square raised cosine filter (SRRC). 

**Figure 3:**
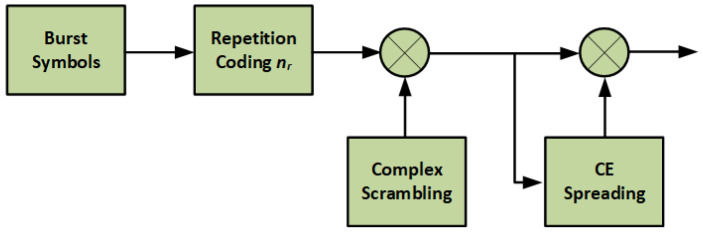
Constant envelope spreading modulator structure.

In the MASSIVE project, an alternative modulation/spreading approach was implemented based on the generation of a quasi-constant envelope signal, as proposed in [11] and investigated in [9]. The proposed structure is shown in Figure 3.

According to [11], there are up to four CE spreading sequences which are evaluated off-line and stored, hence there is one sequence per each possible phase difference among the QPSK modulated symbols. The phase differences between consecutive QPSK symbols are used to select one of the four CE spreading sequences for the chips at the edge of each spread symbol so that the phase transitions from one symbol to the next remain continuous. This will avoid any abrupt phase transition, hence resulting in a quasi-constant signal envelope. From the transmitter perspective the quasi-constant envelope would allow the power amplifier to operate close to the saturation point which would result in a higher power efficiency without introducing any spectral side-lobes or distortion. At the receiver, there is no modification required since the alternation of the spreading sequence at the edge of each symbol can be neglected especially for spreading factors larger than 16. 

### 3.3. MASSIVE Waveform Definition

Capitalizing on the technical innovations described above (i.e., the support of QPSK modulation, TDM between pilot and data symbols and CE spreading) with respect to the heritage waveforms of [5], a new family of waveforms named MASSIVE waveforms were designed, implemented and tested in the project [10]. 

The MASSIVE waveforms parameters were designed to support, in particular, the satellite IoT scenarios as described in previous sections. These parameters include the support of a lower chip rate (160 kcps), several spreading factors (16, 32, 64, 128 and 256), data packet sizes (38 or 150 byte) and coding rates (1/3, 1/2, 2/3).

Table 1 summarizes a sub-set of such waveforms as already tested in the MASSIVE test-bed. The modulation and coding (ModCod) pairs and spreading factor (SF) is specified for each case

## 4. ME-SSA Receiver Design

In this section, first a general overview of the ME-SSA receiver is provided in Section 4.1. Then, Section 4.2 and Section 4.3 focus on the innovative blocks with respect to the conventional E-SSA receiver, i.e., the advanced PS, to dealing with impairments typical of non-geostationary (GEO) satellite scenarios, and the burst demodulator based on the MSD approximation of the linear MMSE detector, to increase the overall throughput performance, respectively.

### 4.1. Recevier Block Scheme Overview

**Figure 4:**
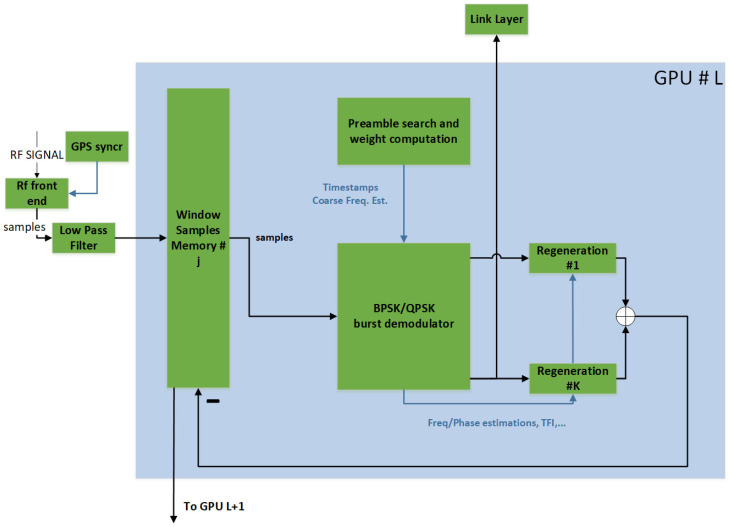
ME-SSA receiver block scheme executed on the generic L*th* GPU (i.e., GPU #L).

Figure 4 shows an overview of the physical layer of the MASSIVE receiver architecture. The received input radio frequency (RF) signal consists of the sum of the signals transmitted by several terminals in an uncoordinated manner. Such a signal is downconverted to baseband and low-pass filtered. The samples are stored in a local memory as a sliding window to perform iterative detection and demodulation processing. 

The receiver processing starts with a PS to detect the presence of bursts in the stored memory window. Then, the identified bursts are demodulated. If a burst is successfully demodulated (checked via CRC), it is locally regenerated and subtracted from the memory window, according to the SIC processing of the E-SSA protocol [4], in order to detect and/or demodulate other bursts not detected and/or demodulated at the previous iterations, due to the higher level of MAI as already discussed in Section 1. Hence, at a high level, the ME-SSA receiver performs the same operations as the E-SSA-based receiver. The difference lies in the specific implementation of the preamble searcher and the burst demodulator algorithms, described in the following sections.

### 4.2. Preamble Searcher

The aim of the PS is to detect the presence of one or multiple bursts within the current search window (stored in a local memory at the receiver). This goal is achieved by cross-correlating the received signal with a local replica of the spread preamble, over a time-frequency grid, i.e., formulating a certain number of time and frequency hypotheses at which a burst could be received [14]. If, for a given pair of time-frequency hypotheses, the correlation value is above a given threshold, the burst detection is positive and the PS returns the associated time-frequency hypothesis for a subsequent demodulation attempt.

A trade-off between computational complexity and performance has been undertaken in order to identify the best PS algorithm depending on the scenario, leading to the following conclusions:

- A coherent PS strategy, where the coherent integration time is equivalent to the preamble duration, is sufficient in the case of a GEO satellite,
- A non-coherent PS strategy is instead needed for LEO or MEO scenarios where the Doppler effect has to be taken into account. Here, the preamble is divided into $N_{c}$ contiguous segments, where coherent integration is carried out on each segment. Then, the resulting $N_{c}$ values are combined, according to the differential post detection integration (DPDI) approach [14].

#### 4.2.1. Coherent PS

**Figure 5:**
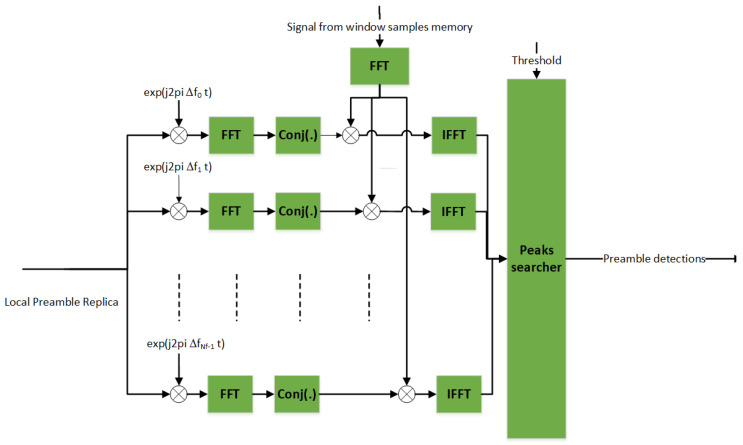
Coherent PS.

In a GEO satellite system scenario and L-Band carrier frequency, the Doppler frequency is negligible, therefore, the maximum frequency uncertainty is mainly due to the local oscillator drift. Assuming a 1 pulse per million (ppm) frequency drift at 1.5 GHz carrier frequency, the maximum frequency uncertainty is *f_max_* = 1.5 kHz. The number of frequency hypothesis is set in a way to guarantee a correlation loss (evaluated as [sin(π*δfT_coh_*)/(π*δfT_coh_*)]^2^, where *δf* (Hz) is the frequency offset error and *T_coh_* (s) the coherent integration time [15]) of less than 0.5 dB (typical value), given the coherent integration time. Considering that the chip rate is $R_{c}=160\;$ kchips and the preamble length is 96 symbols, the number of frequency hypotheses ranges between 77 and 1239, depending on the spreading factor. Indeed, for a fixed chip rate, the higher the spreading, the longer the preamble duration and hence coherence time, thus, the higher the number of frequency hypotheses to have the same correlation loss. These values do not cause any memory/processing limitation at the receiver. The actual coherent PS implementation is based on the cross-correlation via fast fourier transform (FFT) to efficiently test simultaneously more delay hypotheses for each frequency hypothesis. The block scheme is depicted in Figure 5.

#### 4.2.2. Non-Coherent PS

In the LEO (or MEO) case, coherent PS algorithms are not suitable for the following two reasons.

Firstly, an excessive Doppler shift leads to a large number of frequency hypotheses, considering the coherent integration time that is equivalent to the preamble duration. For instance, considering a LEO satellite at 500 km altitude and satellite visibility at a low elevation angle (e.g., about 10°), the maximum Doppler shift is about 36 kHz at 1.5 GHz carrier frequency. Hence, for the MASSIVE waveform, the number of frequency hypothesis can range between 1858 and 29,729, depending on the spreading factor. The search space could be reduced by limiting the lower limit of the elevation angle. For instance, for elevation angle values above 55°, the maximum Doppler is about 20 kHz, that reduces the number of hypotheses; however, the size of the frequency hypothesis space would still be high.

Secondly, an excessive Doppler rate α, for example up to about 400 Hz/s for a 1.5 gHz carrier frequency and 500 km altitude LEO satellite, would degrade the performance of the coherent PS algorithm. The unknown value of the Doppler rate at the receiver causes a considerable phase rotation among the symbols involved in the coherent integration. Depending on the ratio between coherent integration time and Doppler rate, such rotation can yield a destructive sum and a consequent loss of performance, analogous to the effect of the correlation loss.

**Figure 6:**
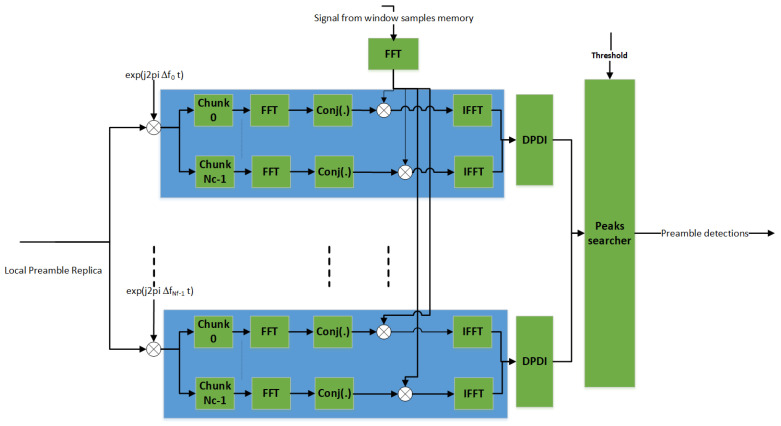
Non–coherent PS.

To mitigate the above-mentioned issues, one solution is to resort to a non-coherent DPDI approach [14] as depicted in Figure 6. This approach reduces the coherent integration time $T_{\mathrm{coh}}$ by a factor of $N_{c}$ compared to the coherent PS solution. For smaller values of $T_{\mathrm{coh}}$, the number of frequency hypotheses will also be reduced, hence making the solution more computationally manageable. Furthermore, the PS becomes robust to the worst-case Doppler rate, since the phase rotation across the symbols in a coherent integration time can be reduced to less than 2°.

### 4.3. Burst Demodulator

**Figure 7:**
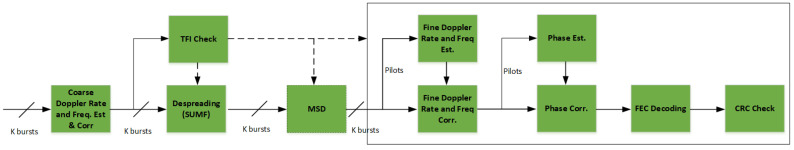
The structure of the burst demodulator.

In this section, we compare the burst demodulator architecture of the E-SSA receiver with a conventional SUMF front-end receiver with the ME-SSA architecture based on the linear MMSE detector implementation. Figure 7 shows the overall burst demodulator functional blocks for both E-SSA and ME-SSA receivers.

The conventional SUMF-based burst demodulator used in E-SSA receivers attempts to demodulate each burst detected by the PS *independently*. As shown in Figure 7, when the SUMF is used, the MSD block is bypassed. It is assumed that K bursts are detected by the PS and the following processing is carried out independently on each of them.

- For each of the K bursts, its samples are extracted by the memory window according to the time estimate of the PS and corrected by the associated frequency.
- A Doppler rate and further coarse frequency estimation on the preamble is carried out.
- The TFI is checked to tune the receiver parameters on the specific burst features.
- Input samples are despread recovering the received noisy symbols.
- Fine Doppler rate, frequency and phase are estimated and corrected by exploiting the pilot symbols.
- Finally, decoding is carried out and CRC is checked to assess if the burst is correctly demodulated.

For the ME-SSA receiver, QPSK modulation is used. In this case, a better spectral efficiency (SE) performance is obtained by resorting to a burst demodulator based on a linear MMSE detector. In the MASSIVE project, a particular version MMSE detector was implemented, known as MSD [9].

**Figure 8:**
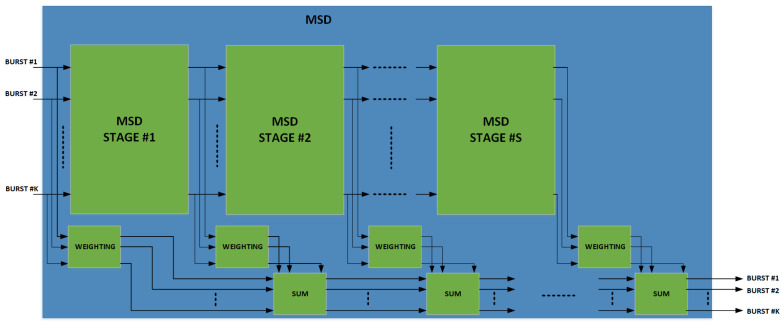
Multistage despreader architecture.

In the case of the ME-SSA receiver, the MSD block depicted in Figure 7 is activated and attempts to *jointly* demodulate all the K interfering bursts detected by the PS. This operation is carried out approximating the theoretical linear MMSE detector through a certain number of identical stages as show in Figure 8. The accuracy of approximation improves by increasing the number of stages. The Linear MMSE detector would require the inversion of the covariance matrix R. The MSD approximates the inverse of the covariance matrix, $R^{-1}$, by a polynomial expansion in R of degree *S*, i.e.,

(1) $$R^{-1}\approx \overset{S}{\underset{n=1}{\sum }}w_{n}R^{n}$$

where $w_{n}$ are the proper weight coefficients. In practice a number of stages *S* equal to 2 and 3 can already give a good approximation of $R^{-1}$. The coefficients $w_{n}$ shall be chosen to approximate the MMSE detector $\sum_{n=1}^{S}w_{n}R^{n}≃{\left(R+N_{0}I\right)}^{-1}$, being $N_{0}$ the noise power. Several methods can be found in the literature for weight computation [16,17]. In this project, the solution derived in [18] was chosen, as it provides a very good approximation of the linear MMSE detector, accounting for the power each symbol is received with, the pulse shaping used, other than the noise power and the system load, i.e., the ratio between the number of interfering bursts and the spreading factor.

**Figure 9:**
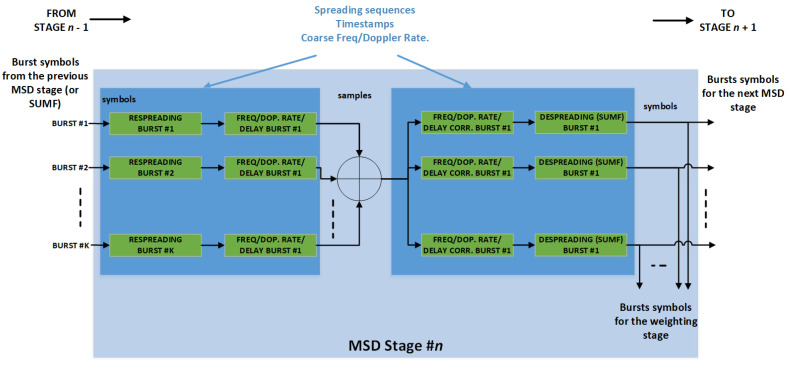
Single block of the MSD, as reported previously in [2].

Let us assume that K interfering bursts have been detected by the PS to be demodulated. First, these bursts are individually despread as in the SUMF case, obtaining, for each of them, the noisy version of the received symbols. Then, the MSD processing takes place, consisting of a sequence of S identical stages depicted in Figure 8. At each stage, the following processing takes place, as shown in Figure 9 and previously reported in [2,9]. 

The K vectors, containing the symbols of the *K* burst as described above, are input to the first stage, where they are re-spread and the estimated time, Doppler rate and frequency offset are restored. In practice, the received spread bursts are regenerated and then summed together. Such composite signal is sent in parallel to K lines, one per burst, where the relevant burst time and Doppler rate and frequency offsets are corrected and then despreading is applied. The K symbol vectors obtained in this manner represent the input to the next stage, which performs the same processing, and so on until all the S stages have been executed. The symbols at the output of each stage are also weighted and summed together. When the last stage has ended, such weighted sum represents the K despread bursts as they were obtained by a linear MMSE detector.

## 5. MASSIVE Test-Bed 

### 5.1. Test Bed for the Evaluation of the Performance

**Figure 10:**
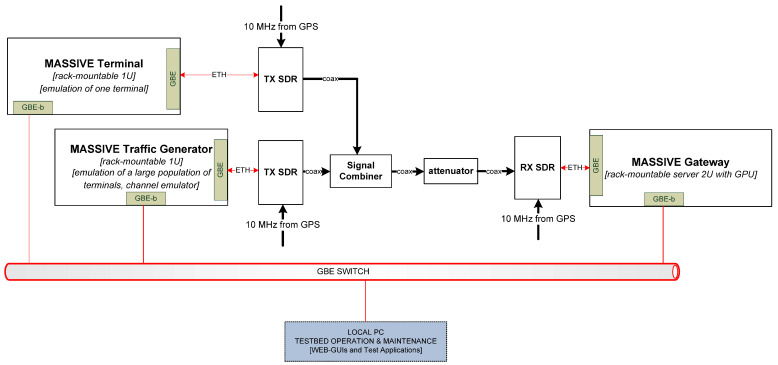
Block diagram of the MASSIVE test-bed.

In order to test the ability of the receiver to manage the signals coming from a number of uncoordinated transmitting terminals, an SDR-based test-bed was designed and integrated in the laboratory. The block diagram in Figure 10 represents the overall structure of the MASSIVE test-bed.

The main components of the MASSIVE test-bed are:

- MASSIVE terminal, a single-unit (1U) rack-mountable server which emulates one terminal,
- MASSIVE traffic generator (TGR), a single-unit rack-mountable server with the aim to emulate the aggregated traffic from a large population of terminals and with the possibility to add a software emulation of the propagation channel,
- MASSIVE gateway, a 2U rack-mountable server equipped with CPU–GPUs with the purpose of emulating the receiver part of the system,
- The SDR radio devices, one per server, which implement the RF front-end.

**Figure 11:**
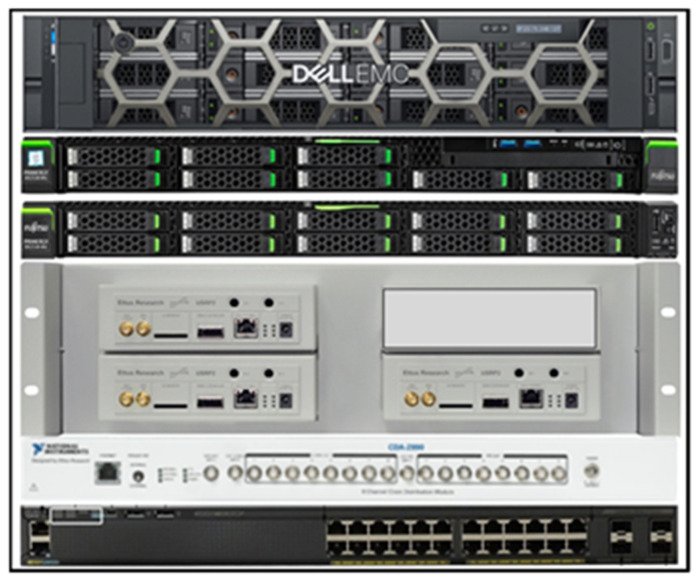
Real rack configuration of the MASSIVE test-bed.

Figure 11 shows the real configuration of the test-bed which was set up in the MASSIVE project and mounted in a rack. As shown in this figure, in addition to the components described above, it included a clock distributor and a network switch.

### 5.2. Receiver Implementation Architecture

Given the nature of the operations in the ME-SSA receiver blocks, the implementation was carried out in a mixed CPU/GPU architecture in order to optimize the performance and to support real-time processing.

The performance of the digital signal processing algorithms performed with general-purpose processors, both CPUs and GPUs, depends on how much the operations can be executed using a vector algebra. In the state-of-the-art CPUs architectures, single input multiple data (SIMD) extensions can be exploited to run batches of floating-point vector operations on contiguous memory, with a single operation that is loaded in the execution pipeline. On the other hand, GPUs are inherently designed to execute the same (or very similar) operations on large batches of memory using a multitude of (logical) threads efficiently.

In the MSD algorithm, all the basic elementary blocks (de-spreading, re-spreading, weight computation, phase rotation, frequency shift) can be implemented in a vector-wise manner; however, in a mixed CPU/GPU application, a decision has to be made on partitioning of the functions among different units. While the final decision depends on fine-level design choices and on the final hardware architecture, the following guidelines can be established:

- Sample-level operations are better suited for implementation on the GPU,
- Symbol-level operations are better suited for implementation on the CPU.

While the copy, typically occurring via the PCIe bus, happens with a relatively high throughput, in the order of hundreds of GB/s, the copies are scheduled in a lazy manner by the GPU driver to achieve an uninterrupted flow of operations. This leads to unpredictable latencies in the digital processing chain. Therefore, the trade-off between copying memory back and forth between the CPU and GPU for faster execution in the GPU while introducing unpredictable latency has been studied, but it needs further careful investigations.

At a system level, the MSD operations cannot be executed all together with a single command in an ideal processing pipeline. This is due, mainly, to the asynchronous nature of the chosen transmission protocol. The incoming asynchronous bursts overlap only partially in time. The weight computation in Equation (1) depends on the number of overlapping bursts. Therefore, it is required to iterate at every boundary between bursts, preventing a massive parallelization. The actual number of boundaries for each sample window increases with both the throughput and the spreading factor. Therefore, two fundamental trade-offs exist:

- First, as the throughput increases, the advantages of utilizing MSD become more prominent, but processing is more demanding and the MAI due to the cancellation residual is also increasing,
- Second, as the spreading factor increases, the use of vector operations becomes more beneficial because of the burst sizes, therefore, the associated contiguous memory becomes bigger, but there are more burst boundaries per sample window, leading to more operations needing to be performed per second.

Finally, the GPU memory could be a limiting factor to be considered as part of the implementation trade-offs. The CPU memory size is not a major constraint, since modern CPUs/systems allow for a large memory. On the contrary, all operations executed by the GPU must reside in the internal memory of every GPU card. Compared to that of CPUs, the available memory of GPU cards is much more limited and not easily upgradable. It is therefore of paramount importance that all data structures are initialized before the digital processing starts. This is because allocating new memory in both the CPU and the GPU is an expensive operation that may lead to an unpredictable latency. Therefore, as part of the MSD operation, a balance between the sample window size and the number of maximum branches executed concurrently should be drawn. These two quantities both demand an increase in memory requirements, and both are required to reach accurate demodulation results.

The physical layer demodulator implemented in MASSIVE is composed of multiple GPUs (10 in simulation for benchmarking purposes and 6 in the used test-bed). Each GPU is used to perform a loop of the SIC algorithm.

## 6. Simulation Results

This section presents simulation results based on laboratory tests carried out using the testbed described in Section 5.1, where satellite IoT terminals (either from the MASSIVE TGR or terminal) transmitted bursts according to the ME-SSA protocol using one of the MASSIVE waveforms defined and implemented in the project (see Table 1). The aggregated traffic was then received and demodulated via the ME-SSA receiver described in Section 4 at the MASSIVE Gateway. 

First, Section 6.1 validates the implementation of the new algorithms and waveforms in the test-bed, discussing also the causes of the implementation loss in the proposed real-time ME-SSA receiver compared to the theoretical results in the literature, based on a non-real time receiver with perfect and full knowledge of the interfering scenario.

Section 6.2 provides results in different a scenario of interests, considering the case of power spreading across bursts, the application to non-GEO satellite scenarios and the adoption of a QPSK modulation with an SUMF only receiver. 

### 6.1. Validaton of the MASSIVE Test-Bed

#### 6.1.1. Validation of MASSIVE Waveforms

**Figure 12:**
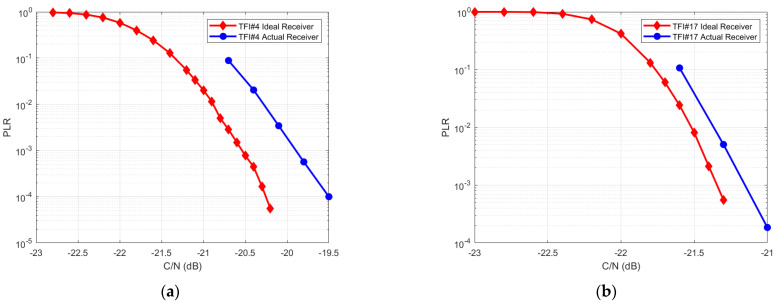
(**a**) Implementation loss less than 1 dB at PLR = $10^{-3}$ for TFI#4; (**b**) Implementation loss less than 1 dB at PLR = $10^{-3}$ for TFI#17.

One of the requirements to validate the implementation was the receiver performance in terms of packet loss ratio (PLR) as a function of the carrier-to-noise ratio (C/N) in the additive white Gaussian noise (AWGN) channel. To this end, the PLR vs. C/N curves obtained by using the actual receiver, where all the estimation algorithms were enabled, were compared with the theoretical curves, where ideal perfect estimators were assumed. The resulting implementation loss was fixed to 1 dB by requirement in the frame of MASSIVE. This was fully achieved, as shown in Figure 12 for waveforms identified by TFI#4 and TFI#17.

#### 6.1.2. Validation of the ME-SSA Receiver and Implementation Loss Analysis

One of the main requirements of MASSIVE is to show the gain in terms of aggregated SE (in bit/s/Hz) or, equivalently, throughput (in pkts/s) of the MASSIVE solution, which is based on MSD, with respect to solutions making use of an SUMF-based receiver.

The MASSIVE receiver (MSD-based) showed performance gains with respect to the SUMF-based receiver for all tested MASSIVE waveforms. For TFI#25, the performance gain, measured in terms of the aggregate throughput, was about 50% whereas for the other MASSIVE waveforms the experienced gain was less than 50%, as shown in Table 2. This was traced down to an imperfect optimization of the demodulation parameters and some hardware limitations.

During the benchmarking and testing activities, the performance of the burst demodulator based on the MSD algorithm was evaluated in the first instance in a stand-alone mode, in order to exclude any bugs in the software implementation of the MSD block only. This test gave performance outcomes similar to the ideal conditions used in [9], where the results were obtained assuming perfect knowledge of all and only interfering bursts, i.e., assuming a perfect PS, and no real time constraints. Therefore, that outcome was considered as a reference to evaluate the performance of the receiver when all its blocks are enabled. 

**Figure 13:**
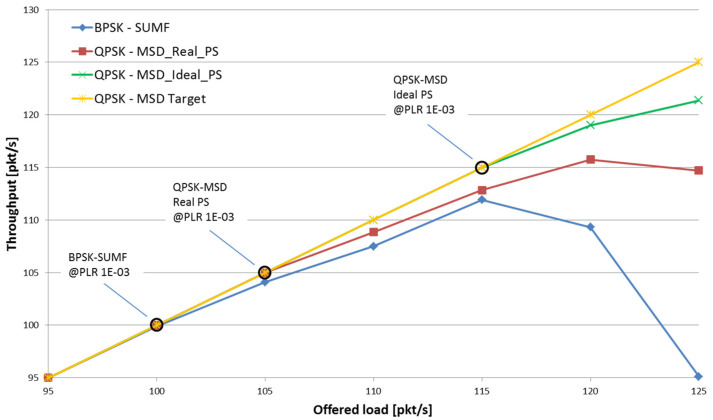
Comparison of Ideal PS vs. Real PS.

Tests were performed in two conditions: (i) an ideal PS and (ii) a real PS. As shown in Figure 13, in the latter case, as expected, the performance was worse than the reference case. Even with a perfect knowledge of the burst positions (an ideal PS) the performance did not coincide with the reference case.

An in-depth analysis was carried out to identify the causes of performance degradation in both cases. The results can be summarized as follows. Specifically, three main causes emerged, one was due to the intrinsic behavior of the real PS, while the other two were instead related to the receiver’s real-time operation constraints, hence affecting both the real and ideal PS results. 

Let us start from the first cause, related only to the use of the real PS. An adaptive threshold $\eta_{i}$ was set to determine which power peaks of the cross-correlations in the current i-th observation window were to be considered as preambles. In the conventional SUMF receiver, the adaptive threshold value depends on the past history of the incoming signals as:

(2) $$\eta_{i}=\max\left(\zeta \cdot \rho_{i},\;0.5\cdot \eta_{i-1}\right)$$

where *ζ* is a constant value depending on the target false alarm rate and number of frequency hypothesis in the PS, while $\rho_{i}$ is the average value of the best cross-correlation peaks per frequency hypothesis.

**Figure 14:**
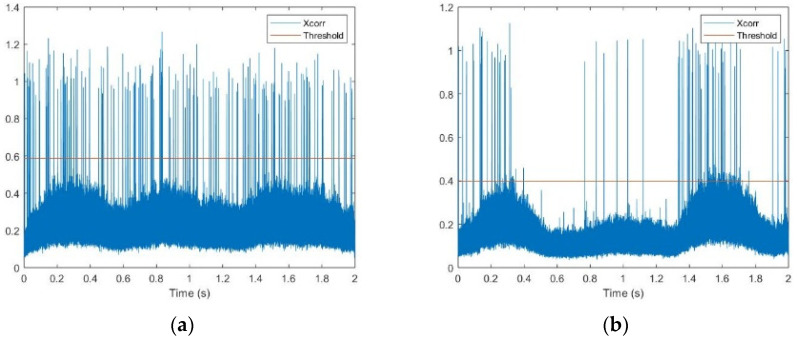
(**a**) PS cross-correlation values over a time-interval before first SIC loop; (**b**) PS cross-correlation values over a time-interval before second SIC loop.

This method in the SUMF case works well as the miss-detection (MD) probability is several orders of magnitude lower than that of the false-alarm (FA) probability. The FAs have instead little or no impact on user performance, as they are discarded following the CRC failure, prior to any burst reconstruction and cancellation stage. Specifically, with this threshold setting, the FAs increase across the SIC loops, due to the following reason. Looking at the cross-correlation values over a given time interval at the first SIC iteration, as depicted in Figure 14a in the case of a high traffic load, the noise plus interference floor of the cross-correlation was rather constant and the cross-correlation peaks emerged clearly. Then, after the cancellation was completed, such floor was not constant anymore but rather had lower values where more cancellations had been performed. The cross-correlation had higher values where cancellation still had to be performed and thus more bursts’ power was still concentrated. As a result, the threshold value was affected by this behavior and, if it were not high enough, it could detect several FAs in the correspondence of higher floor values (for instance, in the region around 1.6 s in Figure 14b).

In this case, the main source of performance degradation was then the presence of FAs and MDs. Due to the MDs, the MSD algorithm did not operate on all the actual interfering bursts, but only on a subset of them, therefore, only partial information was available when evaluating the weights and estimating the inverse of the correlation matrix via the MSD stages. Furthermore, the FAs actually introduced incorrect information that further degraded the algorithm performance.

In order to limit this problem, a modification of the adapting threshold mechanism was adopted. The threshold was then computed by taking the average of only the half most powerful peaks and taking the max between the current values and 0.99 times the former one, i.e.,

(3) $$\eta_{i}=\max\left(\zeta \cdot \rho_{i},\;0.99\cdot \eta_{i-1}\right)$$

The second cause of performance degradation was identified as the sliding window mechanism and the need for the receiver to work in real-time. Two consecutive sliding (memory) windows had certain overlaps so that bursts that were not fully contained in the preceding window were recovered in the following one. Thus, bursts that were received on the boundary between two consecutive sliding windows may have been detected only in the second window, even though they could also have caused a partial interference to the bursts received in the first window. This is equivalent to a misdetection with the MSD receiver working again with partial information only.

The third cause of the non-optimal performance in the case of the ideal PS was due to the behavior of the scheduler block, which was in charge of receiving the outcome of the PS block and preparing appropriate data structures for the demodulator.

The normal flow of data at the receiver provides the output of the PS in the form of a chunk. A chunk is a data structure made of the samples received by the radio front-end and the bursts starting points identified by the PS. The length of one chunk is not correlated to the simulation duration and is a constant valued of approximately 1 s. The number of chunks the PS asynchronously emits is not constant. They are received by the scheduler which separates the data sample part from the starting point information and prepares two distinct vectors for the demodulation. At this stage the dispatching of the vectors towards the demodulation block takes place with a “dispatch if not empty” logic, meaning that the information is delivered to the demodulator as soon as at least one element is in the vectors. In our case this logic turned out to cause a performance degradation, since a burst may have been delivered to the burst demodulator with a limited number of interfering bursts. Again, an effect equivalent to an MD that negatively impacts by definition the intrinsic ability of the MSD algorithm. This consideration led to carrying out the following activities with the purpose of trading-off the SE performance and real-time requirements:

- The dispatcher logic was replaced with a “dispatch if full” logic. This meant that the scheduler accumulated chunks until a certain size was reached. When the vector was full it was delivered to the burst demodulator block,
- The size of the vectors was under a fine-tuning procedure which had the goal of finding a trade-off between providing a sufficient number of interfering bursts to the burst demodulator based on MSD while not significantly impacting the real-time functioning of the system.

### 6.2. Performance Results

#### 6.2.1. Effect of Power Randomization 

As an evolution of the E-SSA solution, the new demodulation strategy was also based on the key features of the E-SSA protocol, e.g., SIC with power spreading. Indeed, as recalled in Section 1, E-SSA benefits from a power imbalance across the received bursts compared to simple SSA. Even for the ME-SSA-based receiver, it has been confirmed that the power randomization on the uplink improves the efficiency compared to the case of no power spreading. 

This has been proven for all the ModCods and spreading factors defined, as reported below. In particular, the measured PLR was compared in the case of no power randomization—where all the bursts were received with the same C/N values equal to the target value listed in Table 3—and in the case of a burst power randomized in a 5 dB range, i.e., for every burst a random number was drawn from a uniform distribution between 0 dB and 5 dB varying the reference power, and where the target C/N was obtained with power spreading equal to 0. It is to be noted that the test was carried out also with the transmission of two different waveforms (TFI#9 and TFI#5) on-air simultaneously.

#### 6.2.2. GEO and Non-GEO Scenarios

Several tests were carried out in a GEO scenario (with a negligible Doppler shift and no Doppler rate) and adding a Doppler shift (up to ±10 kHz) and Doppler rate (up to 400 Hz/s) in order to approximate as much as possible a LEO condition.

The MSD-based receiver showed good performance in demodulating most of the defined waveforms in both a GEO and LEO environment. Some problems arose with the most challenging waveform TFI#33, denoted by the highest spreading factor value (256), only in the LEO environment. Indeed, the combination of Doppler shift and Doppler rate coupled with the long burst size, made the demodulation highly time consuming and resource intensive; however, it should be noted that the demodulation results outperformed the benchmark for all the waveforms except for the TFI#33 in the presence of a Doppler shift-only (±20 kHz) and/or Doppler rate-only (400 Hz/s).

**Figure 15:**
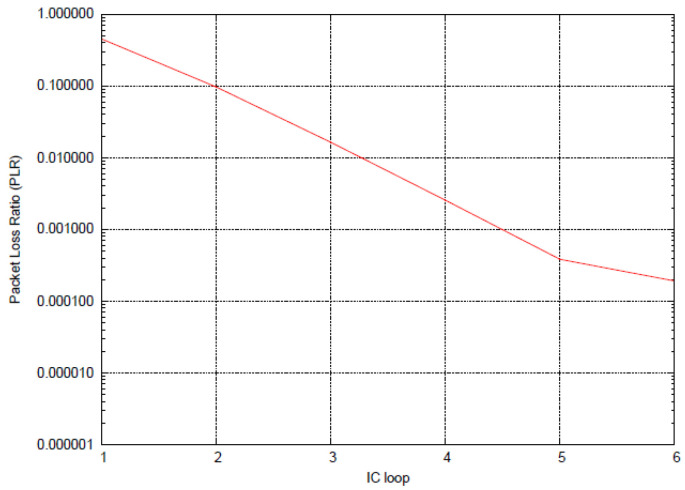
PLR trend with SIC, case TFI#25.

Thanks to a channel emulator that was integrated as part of the traffic generator in the test-bed, the demodulation performance was evaluated even in the case of mobile terminals with a speed up to 130 km/h in both GEO and LEO scenarios. The test was successful, in terms of handled traffic load and PLR at the working point, with the application of a Pérez-Fontán mobile fading channel [19]. Figure 15 shows the trend of the PLR which reached the 10^−3^ threshold after four IC loops.

#### 6.2.3. QPSK with SUMF Receiver

**Figure 16:**
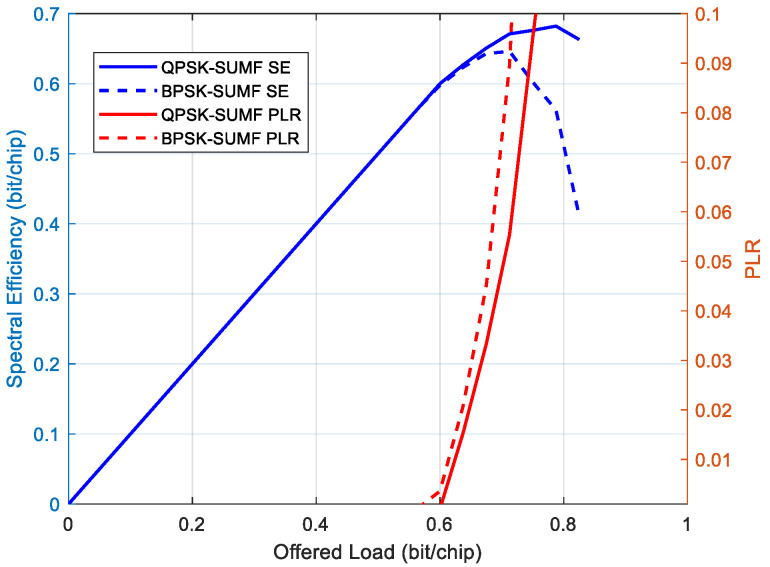
SE and PLR of QPSK with SUMF vs. BPSK with SUMF, minimum C/N = 0 dB and power spreading 3 dB.

In this section, we show that the QPSK modulation with SUMF could also be employed and outperform the BPSK in terms of maximum achievable spectral efficiency results. Indeed, there were cases, depending on the minimum C/N and power spreading, where the SE performance obtained with the QPSK modulation plus SUMF that were quite close or even better than those obtained with the BPSK modulation. Figure 16 shows such an example comparing the SE performance of the MASSIVE waveform with TFI#25, which employed the QPSK modulation, with a waveform sharing the same features but for using the BPSK modulation.

In such scenarios, two opposite cases can be defined, depending on the needs, in which a QPSK with SUMF still represents a viable solution compared to BPSK. The SE can be written as:

(4) $$\zeta =N_{\mathrm{STT}}\cdot \frac{r_{b}}{r_{c}}\;\left[\mathrm{bit}/\mathrm{chip}\right]$$

where $N_{\mathrm{STT}}$ is the number of simultaneous transmitting terminals, $r_{b}$ is the per-terminal bit rate and $r_{c}$ the chip rate. Hence, if $\zeta^{\left(\mathrm{BPSK}\right)}≃\zeta^{\left(\mathrm{QPSK}\right)}$, where $\zeta^{\left(\mathrm{BPSK}\right)}$ and $\zeta^{\left(\mathrm{QPSK}\right)}$ are the aggregated throughput values obtained with BPSK and QPSK plus SUMF, respectively, for the same chip rate and coding rate values, then the following cases hold.

- Use Case #1: QPSK modulation can be chosen in order to double the data rate per-terminal, at the expense of reducing the number of simultaneous transmitting terminals by half.
- Use Case #2: BPSK modulation can be chosen in order to double the number of simultaneous transmitting terminals, at the expense of reducing the data rate per-terminal by half.

## 7. Concluding Remarks

The work carried out during the MASSIVE project focused on the definition of new waveforms based on the features of the ME-SSA protocol (QPSK modulation and regular time-domain pilot symbol insertion). The work also led to the implementation and testing of quasi-constant envelope spread spectrum signals as well as the implementation of the MSD algorithm in a real-time ME-SSA receiver.

The ME-SSA receiver implementation covered the following important aspects:

- The PS, resilient to non-GEO scenario environments, and its adaptive threshold mechanism to better handle MDs and FAs in the case of an MSD demodulator,
- The single stages of MSD, which is the most resource demanding block of the receiver chain, in order to optimize their behavior as much as possible, trying not to increase the complexity beyond measure, while remaining adherent to the specifications,
- The weight calculation block, in order to find and implement the algorithm to calculate the weights to best approximate MMSE in the MSD approach.

The real-time implementation of algorithms revealed many challenges to reach the target performance results obtained by the software simulation in [9]. In particular, implementation issues of the real-time preamble search and associate misdetection and false alarms were investigated in depth.

To this end, the logic of some heritage blocks of the demodulation chain was not optimized to match with MSD requirements at the beginning, and many optimizations were performed on algorithms and constituent blocks of the designed receiver. For instance, the logic of the scheduling mechanism used to feed the demodulator with detected data chunks was modified from a “dispatch if not empty” to a “dispatch if full” logic.

As a concluding remark of the undertaken project, it is confirmed that the adoption of the ME-SSA solution at the receiver, based on evolved PS, MSD and SIC sub-blocks, increases the spectral efficiency or aggregated throughput, compared to that of the E-SSA receiver. This is achieved at the cost of an increased complexity, a higher resource demand and latency (especially for the waveforms with a higher spreading factor, e.g., 128 and 256). 

Overall, a trade-off between the spectral efficiency improvement and the drawbacks may depend on the use case and system parameters to be considered. It is also confirmed that the power spreading of the received packets has a positive impact on the spectral efficiency improvement even in the case of an MSD-based receiver.

In conclusion, the implementation of the ME-SSA solution has shown that it is possible to achieve spectral efficiency gain with reference to the E-SSA solution. This translates into the possibility of accommodating a higher traffic load and number of uncoordinated terminals that can be managed by a satellite gateway. 

## 8. Future Perspectives

The MASSIVE project led to the definition of an air-interface suited for the user up-link with GEO Ku- and Ka-band and LEO scenarios. In particular, the robustness of the air-interface defined in MASSIVE allows the possibility to operate low traffic and low duty cycle terminals with low gain flat (e.g., patch array) antennas with Ku and Ka band GEO satellites. Such air-interface, named IURA (IoT Universal Radio Access), is based on the E-SSA adaptation defined in MASSIVE on the return link, which is able to work at very low C/N (below −20 dB) thanks to the processing gain provided by the high spreading values (up to 256). These values enable the possibility to equip the terminals with small low gain antennas, e.g., an 8 × 8 patch array, and still be able to operate in typical GEO Ku and Ka band scenarios. This, combined with the offered data rates, ranging from hundreds of bit/s to some tens of kbit/s with larger flat antennas, and the possibility to manage a very high number of devices which have to sporadically transmit few data packets, makes it suitable to realize a simple and low-cost terminal for satellite IoT and medium data-rate scenarios.

## Figures and Tables

**Table 1:** Return Link Waveforms.

WF ID	ModCod	SF
TFI#25	QPSK1/3	16
TFI#9	QPSK1/3	32
TFI#4	BPSK1/3	64
TFI#5	QPSK1/3	64
TFI#7	QPSK2/3	64
TFI#6	QPSK1/2	64
TFI#17	QPSK1/3	128
TFI#33	QPSK1/3	256

**Table 2:** Examples of throughput gain.

WF	SF	MODCOD	C/N [dB]	MAC Load [pkt/s] @PLR = 10^−3^ SUMF	MAC Load [pkt/s] @PLR = 10^−3^ with MSD	Percentage Improvement
TFI#25	16	QPSK1/3	−12.0	79	119	50.63%
TFI#9	32	QPSK1/3	−15.1	102	124	21.56%
TFI#7	64	QPSK2/3	−11.8	175	210	20.00%

**Table 3:** Measured PLR comparison with or without power randomization.

WF	SF	MODCOD	C/N [dB]	Measured PLR No Power Randomized	Measured PLR Power Randomized
TFI#25	16	QPSK1/3	−11.4	0.03553	0.00338
TFI#9	32	QPSK1/3	−15.4	0.08752	0.00000
TFI#4	64	BPSK1/3	−19.9	0.57831	0.00005
TFI#5	64	QPSK1/3	−16.6	0.54291	0.00054
TFI#7	64	QPSK2/3	−11.2	0.00764	0.00155
TFI#6	64	QPSK1/2	−13.7	0.00084	0.00064
TFI#17	128	QPSK1/3	−21.1	0.16193	0.00043
TFI#33	256	QPSK1/3	−23.2	0.01181	0.00776
TFI#9 and TFI#5	32	QPSK1/3	−10.0	0.23406	0.00155
	64				

## Data Availability

Not applicable.
